# Citation counts and journal impact factors do not capture some indicators of research quality in the behavioural and brain sciences

**DOI:** 10.1098/rsos.220334

**Published:** 2022-08-17

**Authors:** Michael R. Dougherty, Zachary Horne

**Affiliations:** ^1^ Department of Psychology, University of Maryland, College Park, MD, USA; ^2^ Department of Psychology, University of Edinburgh, Edinburgh, UK

**Keywords:** citation counts, bibliometrics, research quality, open science

## Abstract

Citation data and journal impact factors are important components of faculty dossiers and figure prominently in both promotion decisions and assessments of a researcher’s broader societal impact. Although these metrics play a large role in high-stakes decisions, the evidence is mixed about whether they are strongly correlated with indicators of research quality. We use data from a large-scale dataset comprising 45 144 journal articles with 667 208 statistical tests and data from 190 replication attempts to assess whether citation counts and impact factors predict three indicators of research quality: (i) the accuracy of statistical reporting, (ii) the evidential value of the reported data and (iii) the replicability of a given experimental result. Both citation counts and impact factors were weak and inconsistent predictors of research quality, so defined, and sometimes negatively related to quality. Our findings raise the possibility that citation data and impact factors may be of limited utility in evaluating scientists and their research. We discuss the implications of these findings in light of current incentive structures and discuss alternative approaches to evaluating research.

## 1. Introduction

Researchers and university administrators often assume that journal impact factors (JIFs) and citation counts are indicators of research quality (e.g. [1,2]). This assumption seems plausible: high-impact journals may seem to have a more selective and rigorous review process, thereby weeding out lower-quality research as a consequence. One might also view citation counts as reflecting something akin to the wisdom of the crowd whereby high-quality research garners more citations than low-quality research. One need not look far to see these assumptions on display: universities often promote bibliometric indices such as citation counts and journal impact factors as indices of ‘impact’ or ‘quality’, academic departments use these metrics for important decisions such as hiring, tenure and promotion, and science journalists promote research from high-impact journals. It is also common for authors to equate impact factors and citation counts with quality [2,3]—an assumption that appears in university promotion and tenure policies [1]. For instance, in their review of promotion policy documents, McKiernan *et al.* [1] found that the vast majority of institutions that include impact factors describe them as positive indicators of quality, significance, importance or impact. The inclusion of these metrics in high-stakes decisions starts with the assumption that there is a positive and meaningful relation between the quality of one's work on the one hand, and impact factors and citations on the other. This raises the question, are we justified in thinking that high-impact journals or highly cited papers are of higher quality?

Before proceeding, it is important to note that citation counts and JIFs are often treated as variables to be maximized, under the assumption that high citation counts and publishing in high-impact journals demonstrate that one's work is of high quality. This view implicitly places these variables on the left-hand side of the prediction equation, as if the goal of research evaluation is to predict (and promote) individuals who are more likely to garner high citation counts and publish in high-impact-factor journals. This view, whether implicitly or explicitly endorsed, is problematic for a variety of reasons. First, it neglects the fact that citation counts themselves are determined by a host of factors that may be unrelated to research quality, or for that matter even unrelated to the science being evaluated [4–6]. For instance, citation counts covary with factors such as the length of the title and the presence of colons or hyphens in the title [7,8] and are correlated with other non-scientific variables, such as the size of one's social network [9], the use of social media and the posting of preprints on searchable archives [10–14]. Citation counts also tend to be higher for papers on so-called ‘hot’ topics and for researchers with greater citation-based reputations [15]. Not only are researchers incentivized to maximize these, but it is also easy to find advice on how to game them [16–18].

Second, treating these variables as values to be maximized invites problematic inferences such as inferring that mentorship quality varies by gender simply because students of male mentors tend to enjoy a citation advantage (see the now retracted paper by AlShebli *et al.* [19]), and even perpetrate systemic inequalities in career advancement and mobility due to biases in citation patterns that disfavour women and persons from underrepresented groups [20,21]. Finally, treating citations and impact factors as the to-be-maximized variables may alter researchers’ behaviours in ways that can undermine science [22]. For example, incentivizing researchers to maximize citations may lead researchers to focus on topics that are in vogue regardless of whether doing so addresses key questions that will advance their field.

If we instead think of citation counts and impact factors as predictors of influential and quality science, we can ask whether they are valid proxies for assessing key aspects of the quality of a researcher. To be clear, these metrics are not and cannot be considered *direct* measures of research quality. But of course, addressing this question requires a way of measuring quality that is independent of citation counts. Past work on this topic has primarily addressed the issue by relying on subjective assessments of quality provided by experts or peer reviews. On balance, these studies have shown either weak or inconsistent relationships between quality (broadly defined by the authors) and citation counts (e.g. [23–25]). One challenge in relying on subjective assessments is that their use assumes that the judges can reliably assess quality—an assumption that has been challenged by Bornmann *et al.* [26], who showed that the inter-rater reliability of peer review ratings is extremely poor. Indeed, controlled studies of consistency across reviewers also indicate a surprisingly high level of arbitrariness in the review process (see [27–29]). Peters & Ceci [29], for instance, resubmitted 12 articles (after changing the author names) that had previously been accepted for publication in psychology journals and found that the majority of the articles that had been accepted initially were rejected the second time around based on serious methodological errors. Similarly, Larwence & Cortes [30] reported a high degree of arbitrariness in accepted papers submitted to the *Neural Information Processing Systems* annual conference, widely viewed as one the premier peer-reviewed outlets in machine learning. In this study, a random sample of submissions went through two independent review panels; the authors estimated that 60% of decisions appeared to arise from an arbitrary process. By comparison, a purely random process would have yielded an arbitrariness coefficient of 78%, whereas a process without an arbitrary component would have yielded a coefficient of 0%. If reviewers who are specifically tasked with judging quality cannot agree on the acceptability of research for publication, then it is unlikely that impact factors or citation counts, which themselves are dependent on the peer review process, are reliable indicators of quality.

Several issues have been raised with the use of bibliometrics for faculty evaluation and the incentive structure for appointments, tenure and promotion. First, there is growing concern about the *improper* use of bibliometrics when evaluating faculty. This concern has been expressed by several scholars [4,31–33] and has been codified in the San Francisco Declaration on Research Assessment (DORA)—a statement that has been endorsed by hundreds of organizations, including professional societies such as the Association for Psychological Sciences. Second, several recent analyses challenge traditional orthodoxy that bibliometrics are valid indicators of research quality. For instance, Fraley & Vazire [34] and Szucs & Ioannidis [35] report a moderate *negative* correlation (approx. −0.42) between impact factor and statistical power in the domains of social psychology and cognitive neuroscience, respectively (see also [36,37]). Despite these results and repeated calls for scaling back the use of bibliometrics in research evaluation, their use is still widespread in evaluating faculty job candidates and promoting them on the basis of their *research trajectory* [1].

The use of impact factors and citation counts as indices of a scholar’s future performance also highlights that these metrics are not only used retrospectively, they are also intended to be diagnostic of how scholars will perform in the future. One of the principle functions of the hiring and tenure processes is prediction: the goal is to predict (and select) scientists with the most promise for advancing science. Realizing this goal requires both that we have access to valid predictors of future scientific accomplishments and that we use those indicators appropriately. To our knowledge, there is no evidence for either of these claims [22]. As discussed, there are several reasons to think that these metrics do not *predict* quality [4,31–33]. In order for hiring and tenuring committees to establish their usefulness would require formally modelling how these factors accounted for a scholar's future performance. Moreover, even if hiring and tenuring committees actually built such a model, they would need to calibrate their decision-making in accordance with the model. We suspect, again, that this is not done, nor would it be.

According to the view that impact factors and citations are useful for assessing research quality, we would expect reasonable, or even strong, positive statistical relationships with objective indices of research quality. Evidence to the contrary or even evidence of a lack of relationship would suggest that their utility has been overestimated. Existing research already suggests that JIFs and citation counts are given weight beyond how well they could plausibly predict future performance, potentially at the expense of other diagnostic measures. Although quality is a complex construct, there are several factors that we believe are indicative of research quality (though, without question, these indicators are themselves only proxies). These include methodological features of an article such as use of random assignment (when feasible) and the use of appropriate control groups, the representativeness of the participant population, the appropriate reporting and transparency of statistical analyses, the accuracy with which results are reported, the statistical power of the study, the evidentiary value of the data (i.e. the degree to which the data provide compelling support for a particular hypothesis, including the null hypothesis) and the replicability of research findings. Many indicators of research quality can be assessed only with close inspection by domain experts (e.g. [23–25]), but some others can be identified using automated data mining tools.

In this paper, we take the latter approach by examining how citation counts and JIFs relate to three aspects of research quality: (i) accuracy of reporting statistical conclusions as measured by the number of errors in the reporting of statistics in a paper, (ii) the strength of statistical evidence provided by the data, as defined by the Bayes factor (BF) [38] and (iii) the replication of empirical findings. Inasmuch as impact factors and citation counts are measures of research quality, we would expect that papers in higher-impact journals and highly cited papers should ‘pass’ some key diagnostic checks; for instance, some indicators of problems or virtues in a paper may be the presence (or absence) of reporting errors, the strength of evidence provided by their data and the independent replicability of the findings. For citation counts and impact factors to be useful for research evaluation, at least as they are currently used, we expect that the associations should be reasonably large. The advantages of our approach are that our chosen indices are quantifiable, verifiable, transparent and not reliant on subjective evaluations of reviewers who may not agree about the quality of a paper.

The analyses reported herein bring together three data sources to address whether citation counts and JIFs have as much utility for assessing research quality as how they are used in practice. These data sources include a dataset used in a large-scale assessment of statistical reporting inconsistencies across 50 845 articles in behavioural science and neuroscience journals published between 1985 and 2016 [39] and 190 replication studies from a collection of repositories (see [40]). Each dataset was merged with the 2017 JIFs and/or article-level citation counts. The raw data and code used to generate our analyses and figures are available on the Open Science Framework: https://osf.io/hngwx/.

There are benefits and limitations that must be acknowledged when using this kind of ‘big data’ approach. Large datasets afford the possibility of detecting even small effects. We expect that citation counts and impact factors are over-weighted, but if these metrics are even relatively weak predictors, that might provide some reason for optimism about their use. However, there is one key limitation that must be acknowledged at the outset. To some extent, we sacrifice some fidelity in distinguishing between statistical tests that reflect more ‘central hypotheses’ tested by researchers, versus hypotheses that reflect more ‘peripheral hypotheses’.

It may be tempting to assume that this limitation of our research indicates that—at the outset—our results may be quite limited because they do not focus on a paper’s central hypotheses. However, a paper is not encapsulated by a single or even a few hypothesis tests. Rather, a paper is the conjunction of the tests authors decided *ought* to be reported to provide the necessary details for the reader to understand the phenomena of interest; authors do not report tests at random. So, even if a given test does not represent the most central prediction of a study, the test reported should nonetheless be *accurate* and have *evidential value*.^1^

## 2. Analytic strategy

Throughout, we perform a combination of Bayesian multi-level modelling to estimate effects and BFs to quantify support for the alternative hypothesis, while including an index of out-of-sample predictive accuracy to aid in assessing the utility of each predictor. Bayesian analyses afford some advantages for fitting models with complex random-effects structures. For all Bayesian models, we set *weakly regularizing* priors in brms [41], which are detailed in our analysis scripts on the Open Science Framework. Crudely put, weakly regularizing priors guide the Markov chain Monte Carlo estimation process but, particularly when a dataset is large, allow the posterior to primarily reflect the data. Posterior predictive checks were conducted for each model to verify model fit. Posterior predictive checking involves simulating the data from the fitted model and graphically comparing the simulations to the observed distribution. This step is useful for diagnosing mis-specifications of the statistical model and ensuring that the model adequately reproduces the distribution. Code for conducting the posterior predictive checks is included in the analysis scripts provided on Open Science Framework.

We had specific questions we sought to answer about the relationship between citation counts, impact factors and measures of research quality, but our analyses were not preregistered and are nonetheless exploratory. Consequently, we checked the robustness of our inferences by changing model parameterizations and prior choices, including or excluding different covariates and group-level effects, and making alternative distributional assumptions (e.g. comparing model fits with Gaussian versus Beta distributions). Throughout the paper, we report the mean posterior parameter estimates and the 95% credible intervals (CIs), but our focus is on characterizing the magnitude of the effects, rather than on binary decisions regarding statistical significance (or not). To assess the usefulness of the various covariates for prediction, we also computed the Bayesian analogue of the *R*^2^ statistic based on leave-one-out (LOO) cross validation [42]. The LOO adjusted *R*^2^ provides a better estimate of out-of-sample prediction accuracy compared to the Bayesian *R*^2^. Finally, we also estimated the BFs for all single-predictor mixed effects models and ordinal analyses. The BF expresses the odds in favour of one model compared to an alternative. Here, we use BF_10_ as an index of the alternative hypothesis relative to an appropriately formed null hypothesis. Unless otherwise noted, we use a point-null hypothesis of no effect, with the prior centred on zero. BFs for mixed effects models used BF functions provided in the brms and bayesresrR [43] packages, whereas the BFs for Kendall’s tau and Mann–Whitney *U* statistics used methods developed in [44] and the Bayesian *t*-tests used the BayesFactor package in R [45]. All Bayesian *t*-tests used the default Cauchy prior with a width parameter of 0.707. This prior places roughly 50% of the mass on effects between −0.707 and 0.707.^2^ A BF_10_ greater than 1 reflects the degree to which the data support the alternative hypothesis, while values less than 1 reflect cases in which the data support the null hypothesis. As a rule of thumb, BF_10_ within the range of 1/3 and 3/1 is interpreted as uninformative, with more extreme values interpreted as providing greater evidence for one hypothesis relative to the other. Although prior authors provide categorical distinctions for BFs of different magnitudes [46], we prefer to interpret BFs as a continuous index of degree of support. We note here that many of the BFs we report are based on large samples, which can result in extremely large values even for relatively small effects. Thus, readers should interpret the magnitude of the BFs within the context of the reported *R*^2^, along with the graphical analyses of the data.

## 3. Question 1: Do article citation counts and journal impact factors predict more accurate statistical reporting?

Hartgerink [39] used statcheck [47], an automated data mining tool, to identify statistical reporting inconsistencies among 688 112 statistical tests appearing in 50 845 journal articles in content areas related to the behavioural and brain sciences. Articles included in the dataset were published between 1985 and 2016. A full description of the data and initial analyses are provided by Nuijten *et al.* [47], but we detail critical aspects of the statcheck dataset which suggest it is a valid measure of our research question. statcheck is an R package that uses regular expressions to extract American Psychological Association (APA) formatted results in a null hypothesis significance testing framework [48]. Among other things, statcheck recalculates the *p*-values within a paper based on the test statistics and degrees of freedom reported with each test. statcheck has been shown to be a reliable indicator of inconsistencies in APA formatted papers. For instance, Nuijten *et al.* [47] calculated the interrater reliability between statcheck and hand coding finding that statcheck and handcoders had an interrater reliability of 0.76 for inconsistencies between *p*-values and reported test statistics and 0.89 for the ‘gross inconsistencies’. We should note that although quite reliable, naturalistic datasets—including the dataset used here—will often entail minor limitations that can affect data quality. These data quality issues can arise for several reasons including errors in converting PDF files to text files, or more systematic errors due to some subfields reporting their analyses in ways which deviate from the American Psychological Association style guide. To consider one example, in reviewing the statcheck data, we observed that many *χ*^2^ statistics appeared to be miscoded. In particular, we observed that statcheck sometimes mis-identified sample size as the degrees of freedom for the *χ*^2^ statistic. This leads to an incorrect computation of the *p*-value. To address this issue, we ran analyses both including and excluding all *χ*^2^ statistics. The reported conclusions are based on analyses that included the *χ*^2^ statistics, but we note here that analyses with the *χ*^2^ statistics converged with those without. The only analyses for which the miscoded *χ*^2^ are included are those reporting decision error rates.

These issues notwithstanding, because of the established reliability of the statcheck dataset, we merged this open-source dataset with article-level citation counts and the impact factors from 2017 to understand the relationship between reporting errors and citation counts and JIFs. Citation counts were obtained using the rcrossref [49] package in R and the impact factors were obtained using the scholar [50] package in R. Although our dataset includes articles from 1985 to 2016, we used only the 2017 JIFs. We chose to use only impact factors from a single year for two reasons. First, review committees evaluating faculty for promotion, tenure and awards generally are not provided JIFs for the year of publication, but rather are provided with the JIF in the most recent year. Given that our ultimate goal is to identify statistical relationships as they might manifest in professional contexts, this approach seems most defensible. However, we recognize that others addressing this problem may make other analytic decisions. Second, and more practically, obtaining JIFs for the full set of articles was not possible, given that existing databases only provided JIFs dating back to 1997. Limiting our analyses to papers published since 1997 would have reduced our dataset by roughly 20%.

Data from each article were then summarized to reflect the number of statistical reporting errors per article and the total number of statistical tests. Articles with fewer than two statistical tests were excluded from our analyses. The motivation for this decisions was again twofold. First, it is rare for an empirical paper to report only a single statistical test. We reasoned that these papers likely reflected commentaries, notes, or editorials and were, therefore, not empirical contributions. Second, articles with fewer than two statistical tests do not allow for computing variability across tests. The final dataset used for our analysis included 45 144 articles and 667 208 statistical tests.^3^

To address the question of whether article citation counts and JIFs predict more accurate statistical reporting, we examined the degree to which citation counts and impact factors predict diagnostics of quality in three ways. First, we examined whether articles containing at least one error were cited more or less than articles with no errors.^4^ Of the 45 144 articles included in the analysis, roughly 12.6% (*N* = 5710) included at least one statistical reporting inconsistency that affected the decision of whether an effect was statistically significant or not. The majority of the decision errors (*N* = 8515) included in these articles involved authors reporting a result as significant (*p* ≤ 0.05), when the recalculated *p*-value based on the reported test statistic (e.g. *t* = 1.90) and d.f. (e.g. d.f. = 178) was greater than 0.05. Only *N* = 1589 consisted of the reverse error, in which a *p*-value was reported as non-significant or *p* > 0.05 when the test statistic indicated otherwise. This asymmetry suggests that these reporting errors are unlikely to be random. Indeed, of the reported tests in which the re-computed *p*-value was *greater* than 0.05 (*N* = 178 978), 4.76% (*N* = 8515) were incorrectly reported as significant, whereas only 0.32% (*N* = 1589) of the re-computed *p*-values that were significant (*N* = 488 154) were incorrectly reported as *non*-significant—a difference that was statistically reliable with strong evidence for the alternative hypothesis (BF_10_ > 1.0 × 10^16^ with a proportion test, beta(1,1)). This result directly replicates prior work by Nuijten *et al.* [47]. More interesting, articles containing at least one reporting error (*M* = 52.1, s.d. = 111.7) garnered more citations than articles that did not contain any errors (*M* = 46.8, s.d. = 98.3). Although the magnitude of the effect is small—indicating that papers with at least one decision error have on average 5 *more* citations than those without errors—the BF provides strong evidence for the alternative hypothesis, BF_10_ = 18.1 (Jeffreys–Zellner–Siow prior two-sample BF, *r*_scale_ = 0.707 [45]). This conclusion was robust to transformation: correcting for skew in citation counts with the log transformation yielded even more convincing evidence that papers with errors are cited more frequently (BF_10_ = 2.4 × 10^8^). Thus, at least by this criterion, and in contrast to the conventional assumption that citations are positively related to quality, this analysis indicates that citations are actually *inversely* related to quality.^5^ Although the above analysis of citation counts neglects the multi-level structure and complexity of the data, we share it because it is likely that people use citation counts in a similar way when inferring research quality, following the general principle of ‘more is better’ without adjusting appropriately for differences across sub-disciplines or accounting for other relevant factors.

Next, we examined the relationship between frequency of errors and impact factor and citations counts using a Bayesian multi-level zero-inflated Poisson model. This model predicted the frequency of errors per paper on the basis of the log of a journal’s impact factor and the log of an article’s citations (+1), controlling for the year of publication and number of authors, with the total number of reported statistical tests per paper included as the offset in the model. Including the offset allows us to appropriately model the error *rate* per paper, while adjusting for the total number of reported statistical tests. Year of publication was included because there have been large changes in APA formatting of statistical analyses since 1985, as well as changes in statistical software over this period of time. We also considered it plausible that the number of authors on a paper could affect error rates in a paper (e.g. more authors may check over the work being submitted and so could reduce the errors in a paper) and so we conducted our analyses with and without the number of authors as a covariate in this model. The inclusion of this covariate did not materially change the conclusion of this set of analyses. Finally, we also classified each journal into one of 14 content areas within psychology (clinical, cognitive, counselling, developmental, education, experimental, general, health, industrial/organizational, law, methodology, neuroscience, social and ‘other’). The classification of the journals into content areas allowed us to control for potential variation in publication practices across areas by including content area as a grouping factor. However, journal was not treated as a grouping factor in the model because it is perfectly co-linear with JIF (i.e. the population and grouping-level effects are identical).

**Figure 1.**
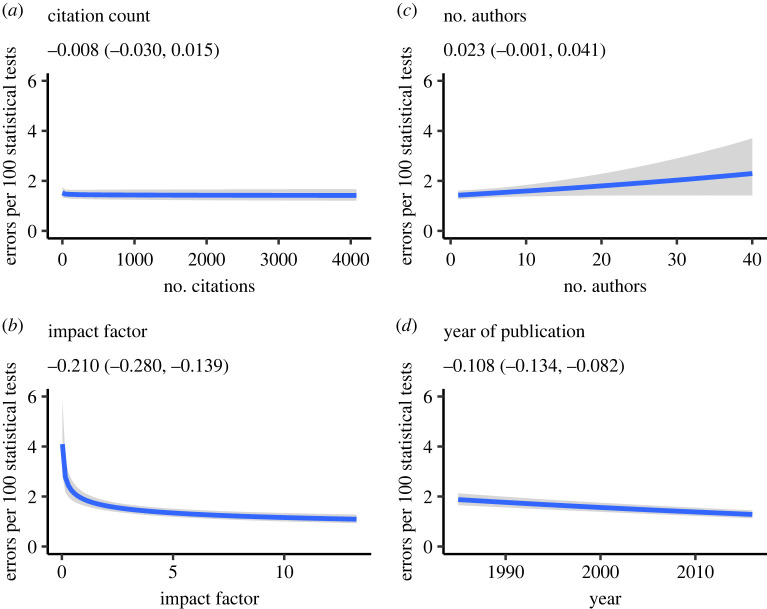
Number of statistical decision errors per 100 tests as a function of (*a*) number of times the article was cited, (*b*) journal impact factor, (*c*) number of authors on the paper and (*d*) year of publication. Shaded regions indicate 95% credible intervals. Analyses based on *N* = 45 144 published articles.

Figure 1 plots the change in error rate (per 100 statistical tests for legibility) as a function of the predictors based on the posterior distribution of the Poisson regression model. As shown, the number of decision errors per 100 statistical tests drops from roughly 4 errors per 100 for the lowest impact factor journals to a little under 2 errors per 100 for the highest impact factor journals, and most of this drop occurs for journals with impact factors between 0 and 2; there is virtually no difference between journals with impact factors greater than 2. Statistically, only journal impact factor *b* = −0.210 (−0.280, −0.139), BF_10_ = 597.57 and year of publication *b* = −0.108 (−0.134, −0.082), BF_10_ = 2.05 × 10^6^ predicted decision errors, with both BFs strongly supporting the alternative hypothesis, indicating 0.23 fewer errors for a one unit increase in the log of JIF, and 0.11 fewer errors for papers published 1 year more recently, respectively.^6^ Analysis of both citation counts *b* = −0.008 (−0.030, 0.015), BF_10_ = 1/1000 and number of authors *b* = 0.023 (−0.001, 0.046), BF_10_ = 1/142 strongly supported the null hypothesis. This later finding is consistent with the work of Veldkamp *et al.* [51].

Table 1 provides *leave-one-out* *R*^2^ and the BF, along with the parameter estimates for each predictor when modelled separately. We include these analyses for two reasons. First, we wanted to verify that the specific findings obtained for the full model were not dependent on the inclusion of the covariates. Thus, the analyses provide a ‘robustness’ test. Second, given that impact factor and citation counts are likely treated independently in faculty evaluations, we wanted to construct models that more closely approximated this type of decision context. For these models, each predictor was entered into the model separately as a fixed effect, including content area as a random effect with total number of reported statistical tests per article controlled by including it as the offset. The BFs were estimated from the posterior distributions using the bayestestR package [43]. All of the analyses included in table 1 support the conclusions from the full model: the evidence for citations and number of authors strongly supports the null, while evidence for JIF strongly supports the presence of a positive relation.

**Table 1.** Parameter estimates, Bayesian leave-one-out (LOO) adjusted *R*^2^ for each predictor modelled separately, and BFs. Each model includes the predictor, with the random-effects factor (area of psychology), and when appropriate the offset, included as control variables. BFs express the evidence in favour of each model relative to a a model with the control variables only. Question 3 includes the year as a random factor as well.

DV	predictor	effect (95% CI)	LOO *R*^2^	BF_10_
*Question 1*				
decision errors	2017 JIF	−0.146 (−0.214, −0.079)	0.021	inf
	citations	0.004 (−0.016, 0.024)	0.021	1/945.4
	authors	0.002 (0.012, −0.024)	0.021	1/861.65
	year	−0.079 (−0.11, −0.06)	0.022	1.48 × 10^7^
*Question 2*				
Bayes factor	2017 JIF	−0.056 (−0.067, −0.044)	0.0049	4.23 × 10^15^
	citations	−0.014 (−0.017, −0.011)	0.0048	4.31 × 10^12^
	authors	−0.010 (−0.014, −0.006)	0.0050	124
	year	0.021 (0.018, 0.025)	0.0045	1.75 × 10^23^
	degrees of freedom	0.190 (0.131, 0.254)	0.0044	1.47 × 10^7^
*Question 3*				
replication	2017 JIF	−0.640 (−1.202, −0.115)	0.022	1/2.31
	citations	0.068 (−0.141, 0.281)	−0.007	1/75.32
	authors	0.158 (−0.187, 0.503)	−0.005	1/38.51

The above findings notwithstanding, it is clear that none of the predictors are of ‘practical’ importance, as evidenced by the *R*^2^. Articles in higher-impact journals are associated with fewer statistical decision errors; however, the fact that the change in error rate only decreases from 4% for journals with impact factors close to zero to a little under 2% for journals with impact factors greater than 10 implies that the relation may not be particularly diagnostic for judging the quality of individual research papers.^7^

The negative relation between the number of errors and the JIF may reflect the quality of the review process (maybe reviewers or editors of higher-impact journals are more likely to catch errors), the strength of the journal editorial team or (perhaps) the possibility that authors are more careful when submitting to higher-impact-factor journals. To address the question of quality more directly, we next evaluate whether the strength of evidence presented in papers varies with impact factors or citation counts.

## 4. Question 2: Do highly cited papers and papers in high-impact-factor journals report stronger statistical evidence to support their claims?

Carl Sagan noted that *extraordinary claims require extraordinary evidence*. From a Bayesian perspective, this can be interpreted to mean that surprising or novel results, or any result deemed *a priori* unlikely, require greater evidence. Many papers published in high-impact journals are considered *a priori* unlikely according to both online betting markets and survey data [52]. Moreover, editorial policies for some higher-impact journals emphasize novelty as a criterion for publication—authors understand that research reports should be ‘new’ and of ‘broad significance’. Assuming high-impact journals are more likely to publish novel and surprising research, then one might also expect these journals to require a higher standard of evidence. One way to quantify the strength of the evidence is with the BF [53]. The BF provides a quantitative estimate of the degree to which the data support the alternative hypothesis versus the null. For instance, a BF_10_ of seven indicates that the data are seven times more likely under the alternative hypothesis than under the null *as those hypotheses are specified*. All else being equal, studies that provide stronger statistical evidence are more likely to produce replicable effects [54], and hold promise for greater scientific impact because they are more likely to be observable across a wide variety of contexts and settings [55,56].

To conduct these analyses, we computed the BFs for all *t*-tests, one degree of freedom *F*-tests and Pearson’s *r* (*N* = 299 504 statistical tests from 34 252 journal articles) contained within the dataset [39] in which the re-computed *p*-value based on the test statistic and degrees of freedom was *p* ≤ 0.05. This procedure allows us to evaluate the general strength of evidence presented by authors in support of their claims, but does not allow us to evaluate cases in which authors’ theoretical conclusions are based on a single statistic among many that might be presented.

The BF was computed using the meta.ttestBF function in the Bayesfactor package in R using the default Cauchy prior (*r*_scale_ = 0.707). This function computes the BF based on a *t*-statistic and sample size. The default prior was used in part because it weights the range of effect sizes that are plausible in behavioural science (i.e. small to medium) more heavily than larger effects and in part because we had no other prior beliefs to justify an alternative to the default. We did not evaluate any alternative priors. We first converted the *F* and *r* statistics to *t* statistics before computing the BF for a given test. Because our automated approach cannot differentiate between within- and between-subjects designs, we computed two separate BFs for each statistic, one assuming within-subjects and one assuming between-subjects. Sample size for the meta.ttestBF function was estimated from the degrees of freedom, with *N* = d.f. + 1 for the one-sample case and *N*1 = *N*2 = (d.f. + 2)/2 for the two-sample case, rounded to the nearest integer. Because the two estimates are highly correlated (*r* = 0.99) and yield similar findings, we report only the results of the two-sample case. It is important to note that while this procedure makes the intercept of our models difficult to interpret, our primary interest was in the *slope parameters*—we sought to understand the relationship between evidential strength and both JIFs and citation counts, *not* the overall or average magnitude of BFs in the papers in our dataset.

Because the distribution of the BFs was extremely skewed, we converted them to probabilities using BF_10_/(BF_10_ + 1) and used Beta regression to analyse the data. This conversion is both natural and justified, and does not alter the interpretation of the data, as it merely re-expresses the BF from the relative probability of two competing models to the probability of a particular model (in our case, the model representing the alternative hypothesis). The Beta regression model is the appropriate approach for analysing data bounded by 0 and 1. The conversion to the probability scale was not possible for a small number (*N* = 188) of the BFs due to their extremity, resulting in a total of 299 316 total observations being used in the final analysis. The BFs reported below for the model predictors were obtained using the bayestestR package [43]. As a robustness test, we also repeated all of the analysis after applying the rank normal transformation to the BF. These analyses are provided on Open Science Framework, and are consistent with the results of the beta regression models reported here.^8^

Under the common assumption that citation counts and impact factors reflect scientific impact or quality, one would expect these indices to be positively correlated with the evidentiary value of the data represented in the publications. However, this does not seem to be the case. In fact, as we demonstrate next, there is a tendency for the evidentiary value of the data to be *weaker* in high-impact journals.

The ‘centrality’ of a hypothesis can only be assessed indirectly in these analyses, but we work from the assumption that tests reported in a paper, particularly those that are statistically significant, will tend to be the tests most central to an author’s argument. Focusing on significant tests, we used citation counts and impact factors as predictors of a test’s BF, controlling for content area, year of publication, number of authors of the paper and the reported degrees of freedom (as a surrogate for sample size).^9^ The magnitude of the BF was negatively related to the JIF, *b* = −0.033 (−0.046, −0.021) and number of authors *b* = −0.014 (−0.018, −0.010). Both of these findings strongly support the alternative hypothesis (BF_10_ = 60.52 and 3.91 × 10^3^ for impact factor and number of authors, respectively). Citation counts also showed a small negative relationship, *b* = −0.004 (−0.008, −0.0001), though the evidence convincingly supported the null hypothesis (BF_10_ = 1/500) in this case. Both year of publication *b* = 0.019 (0.015, 0.023) and degrees of freedom *b* = 0.184 (0.124, 0.248) were positively related and showed convincing support for the alternative hypothesis (BF_10_ = 1.31 × 10^7^ and 1.30 × 10^5^ for year and degrees of freedom, respectively). Table 1 provides the regression coefficients, LOO *R*^2^, and BF for each predictor modelled separately. Again, these models are included as both robustness tests and to examine impact factors and citations counts in particular, but also number of authors, independently as they might be used in tenure and promotion contexts. The BF_10_ values reported in table 1 were computed using the bayestestR package [43].

**Figure 2.**
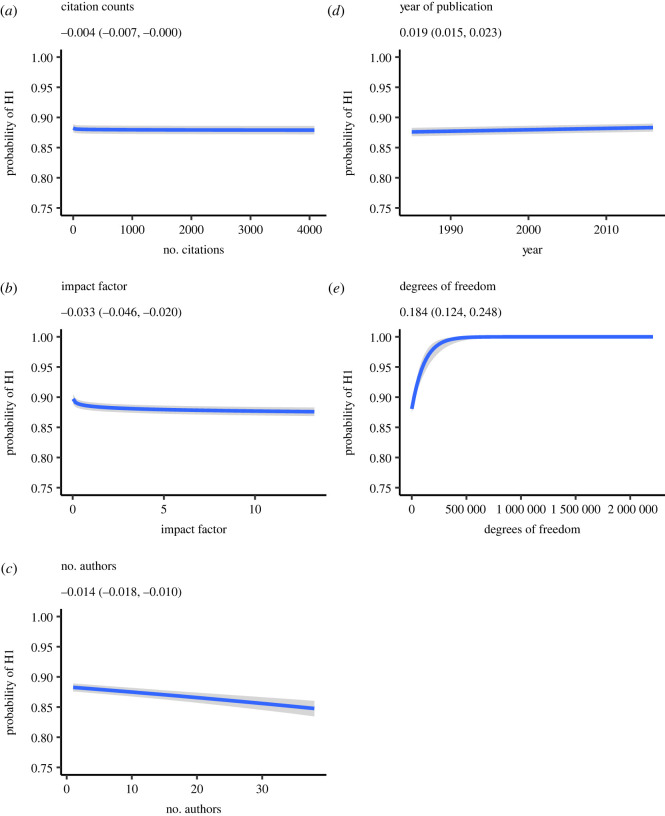
Bayesian estimated posterior probability for *H*_1_ for all *t*-tests and one degree of freedom *F*-tests that were statistically significant at the *p* < 0.05 level (*N* = 299 316) as a function of (*a*) number of times the article was cited, (*b*) 2017 journal impact factor, (*c*) year of publication, (*d*) number of authors on the paper and (*e*) reported degrees of freedom. Shaded regions indicate 95% credible intervals.

Although the BFs for each predictor provide convincing support for each predictor, the magnitudes of the effects are quite small and likely not of much practical relevance. For instance, as shown in figure 2, the estimated probability of *H*_1_ decreased from approximately 0.90 to 0.87 (corresponding to BF_10_ = 9 and BF_10_ = 8, respectively) across the full range of impact factors plotted. Similarly, the probability of *H*_1_ is only marginally higher in papers cited a mere 10 times relative to those cited over 1000. Thus, while the strength of evidence presented in papers is clearly related to JIFs, citations and number of authors, this relation is small. Nevertheless, what should be clear from these analyses is that the use of impact factors and citations as positive indicators of quality is not justified. These indices are, at best, uninformative indicators of research quality (as defined by strength of evidence) and, at worst, misleading indicators in which higher-impact factors and greater citations reflect *poorer* quality.

The small yet convincing negative relationship between evidentiary value and number of authors indicates that papers with greater numbers of authors were also associated with a given test providing less evidentiary value. To speculate, in the absence of preregistered analysis plans, the number of alternative ways the data could be analysed may grow as a function of number of authors, a hypothesis consistent with the findings of Silberzahn *et al.* [57]. In turn, it is possible that weak evidence discovered during extended exploratory data analysis is over-interpreted. However, further research would be needed to confirm this hypothesis. As with analyses of the decision errors, the Bayesian *R*^2^ for these analyses are small, indicating that despite the fact that the results are reliable, none of the predictors account for much variance in the magnitude of the BF (see table 1).

### 4.1. Further exploratory analyses

Another way to look at the data is to model the distribution of *p*-values directly. We next examined the degree to which the magnitude of the computed *p*-value co-varied with impact factors, citation counts and number of authors, controlling for year of publication, number of statistical tests reported for each article, and content area within psychology. We modelled only those *p*-values ≤0.05 under the assumption that these are the most meaningful of authors’ findings. Of the 667 208 total statistical tests included in the dataset, 488 151 of the re-computed *p*-values were less than 0.05. Bayesian beta regression again revealed that both the impact factor *b* = 0.041 (0.032, 0.050) and the number of authors *b* = 00.008 (0.006, 0.011) predicted the magnitude of the reported *p*-values with BFs supporting the alternative hypothesis (BF_10_ = 1.31 × 10^8^ and 3.27, respectively). Journals with higher impact factors and papers with more authors were associated with reporting higher *p*-values (i.e. *p*-values closer to the critical threshold of 0.05), though the BF for number of authors indicates weak evidence. The BF for citation counts indicated support for the null (BF_10_ = 1/36), though the majority of the posterior distribution was negative, *b* = −0.004 (−0.007, −0.002). The BF for year of publication clearly supported a negative relation, *b* = −0.017 (−0.020, −0.014), BF_10_ = 4.53 × 10^9^, suggesting that more recent publications report smaller *p*-values to support their claims. Generally, the effect of the log of the total number of tests included in each paper supported the null hypothesis as well, BF_10_ = 1/16, though again the majority of the posterior distribution was negative *b* = −0.007 (−0.01, −0.003). As a robustness check, we fitted a separate model without controlling for the total number of reported statistical tests. This analysis did not alter our conclusions.

## 5. Question 3: Do citation counts or journal impact factors predict replicability of results?

To address this third question, we used an openly available dataset that included data from eight different replication projects, including the Reproducibility project [58], Many Labs 1 [59], Many Labs 2 [56] and Many Labs 3 [55], a special issue of Social Psychology [60], the Association of Psychological Sciences Registered Reports Repository [61], the pre-publication independent replication repository [62] and Curate Science [63]. Reinero *et al.* [40] collated these data, which also includes the number of citations to each original article, the number of authors on the original article, year of publication and the Altmetric score for the original article. The full dataset curated by Reinero *et al.* [40] is available on the Open Science Framework (https://osf.io/pc9xd/). We merged these data with the 2017 JIFs obtained using the scholar [50] package in R and coded each journal by subdiscipline (clinical, cognitive, social, judgement and decision making, general, marketing and ‘other’).^10^ Of the 196 studies included in [40], we were unable to obtain JIFs for four of the publications; an additional two papers were not coded for replication success. Thus, the total number of studies included in our analysis was *N* = 190, of which 80 (42.1%) were successfully replicated.

A subset of the replications included in this dataset are from the replication project [58] and include a variety of other variables, including ratings of the ‘surprisingness’ of each original finding (the extent to which the original finding was surprising, as judged by the authors who participated in the replication project) as well as citations to the first author of the original paper and prestige of the authors' institution. Our original analyses provided in early drafts of this paper (https://psyarxiv.com/9g5wk/) focused only on the 100 studies included in the repository [58]. We expanded our analysis in March 2021 to include all of the studies reported in [40]. The dataset [58] includes only three journals (*Journal of Experimental Psychology: Learning, Memory and Cognition*, *Journal of Personality and Social Psychology* and *Psychological Science*), and the impact factors for these three journals are completely confounded with content area within psychology. The expanded dataset includes 190 total publications in 27 different journals, thereby enabling us to include impact factor as a predictor in our models.

**Figure 3.**
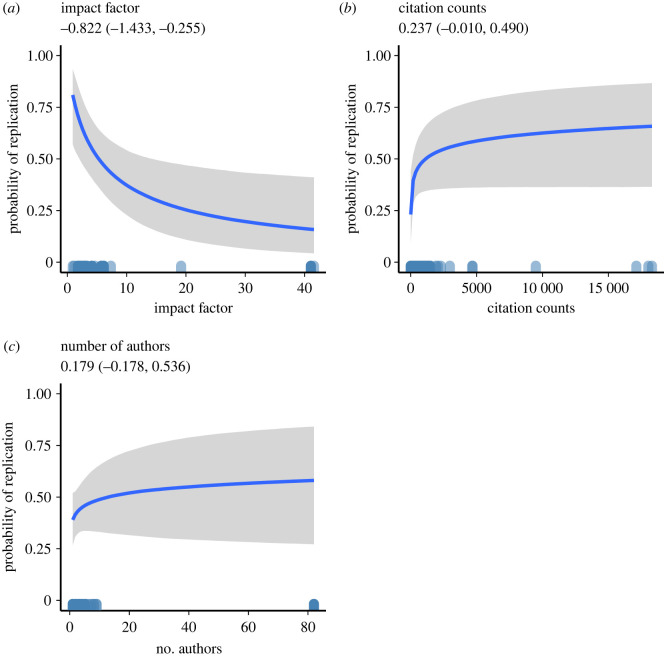
Bayesian estimated posterior probability of replication success as a function of (*a*) 2017 journal impact factor, (*b*) number of times article was cited and (*c*) number of authors on the paper. Shaded regions indicate 95% credible intervals. Ticks on the *x*-axis indicate the distribution of individual cases for the predictor variable.

To examine whether impact factors and citation counts predict replication success (as defined by the original authors who replicated the prior studies), we conducted a Bayesian multi-level logistic regression predicting replication success from the log (impact factor), log (citation count + 1) and the log (number of authors) with year of publication and area within psychology included as a random or grouping factors, using weakly informative priors. Figure 3 plots the Bayesian estimated posteriors. The relation between impact factor *b* = −0.822 95% CI (−1.433, −0.255) and replication success was negative, indicating a 30% decrease in replication success for a one unit increase in the log of a JIF. This is in the opposite direction than would be desired by proponents of their use, though the BF was equivocal, indicating that the data do not differentiate between the null or alternative hypotheses (BF_10_ = 1.5). Analyses of citation counts *b* = 0.237 95% CI (−0.010, 0.490) and number of authors *b* = 0.179 95% CI (−0.178, 0.536) indicated that the data strongly support the null hypothesis in both cases (BF_10_ = 1/13 and 1/34, respectively). As a robustness analysis, we fitted alternative models excluding ‘area’ as a grouping factor, modelling the predictors individually and specifying alternative prior distributions. These models all yielded consistent findings, with the slope of the impact factor consistently negative ranging from −1.03 to −0.53, and the evidence for the effect of citation counts and number of authors showing support for the null. Note that the bulk of the articles used in this analysis were from journals with impact factors less than 10, and that the relation between impact factors and replication success is strongest in this range. An exploratory analysis eliminating the eight articles with impact factors greater than 10 indicated relatively stronger support for the negative relation among this subset of papers, *b* = −1.227 95% CI (−1.990, −0.492), BF_10_ = 6.6.

**Figure 4.**
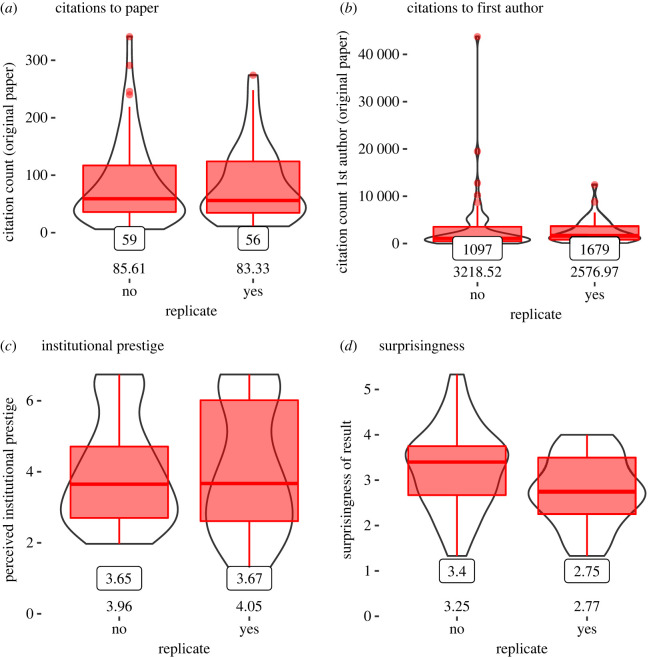
Violin plots of (*a*) number of times each paper is cited, (*b*) number of times the first author was cited, (*c*) rated institutional prestige and (*d*) rated surprisingness of experimental findings split by studies that were and were not successfully replicated. Medians are given by boxed numbers; means are unboxed. Plots are based on the *N* = 100 replication attempts included in [58].

As noted above, a subset of the data was drawn from the repository [58]. This dataset included a number of additional variables that might be viewed as reflective of quality, including institutional prestige, number of citations garnered by the author, as well as number of citations to the original paper. As an index of ‘novelty’, the dataset also includes ratings of *surprisingness*. Figure 4 displays violin and boxplots for the subset of studies in [58], split by whether they were successfully replicated. We conducted a Bayesian logistic regression using number of citations to the original paper, number of citations to the first author, institutional prestige and *surprisingness* as predictors of whether the original finding was replicated, with replication success defined based on whether the replication study was statistically significant. Of these indices, only surprisingness showed a relatively strong relation with successful replication *b* = −0.663, 95% CI (−1.225,−0.121), though there was no clear support for either the alternative or null hypothesis (BF_10_ = 1/1.58). Nevertheless, the observed negative relationship is consistent with other recent work [52,64] showing that effects viewed as having low prior probability were less likely to replicate. Neither the number of citations to the original paper (BF_10_ = 1/1202), *b* = 0.005 95% CI (−0.003, 0.014), nor the number of citations garnered by the first author of the original paper (BF_10_ = 1/131062), *b* = −0.00005 95% CI (−0.0002, 0.00005), nor the institutional prestige of the first author (BF_10_ = 1/67) were predictive of replication success *b* = 0.014 (−0.317, 0.274) among this subset of replications.^11^

The above analyses are consistent with the results presented for Questions 1 and 2, in that neither JIF nor citation counts reflect key aspects of research quality. Table 1 summarizes the key findings for all three questions we sought to address for all of the single-predictor models. If anything, the evidence seems to suggest that higher-impact-factor journals publish work that is *less* replicable consistent with recent research using a smaller, less representative sample [65]. Thus, in regard to the question of whether citation counts or JIFs are *positive* indicators of quality, as defined by replicability, the answer appears to be ‘no’, at least given the data used to address this question.

Altogether, the only reason for optimism for the use of JIFs as a metric for research quality is the slight reduction in decision errors in these journals (Question 1). Though, as indicated elsewhere in our analyses, this optimism should be tempered by the fact that higher-impact-factor journals also tend to publish papers that present less evidentiary support (Question 2).

## 6. Discussion

Goodhart’s Law states that a measure ceases to be a good measure when it becomes a target [66]. Even if citation counts and JIFs were once reasonable proxies for quality (though there is no evidence to suggest this), it is now clear that they have become targets and as a result lack validity as *positive* indicators of faculty performance [67]. On balance, our findings are consistent with this conclusion: in only one case were either impact factor or citation counts a positive predictor of quality. In almost all other cases, there was either convincing evidence that these variables were associated with poorer quality or convincing evidence for the null. Regardless, in *all* cases, the magnitudes of the observed relations suggest that neither citation counts nor impact factors were *meaningfully* related to research quality, as defined by errors in statistical reporting (Question 1), strength of evidence as determined by the BF (Question 2) or replicability (Question 3). The strongest relation was observed for replicability, though this finding should be interpreted cautiously due to the limited nature of the dataset and the uncertainty of the estimates. Though there is evidence in Question 1 that impact factor is inversely related to statistical reporting errors (fewer errors in higher-impact journals), this finding comes with numerous caveats. For example, the magnitude of this relationship was trivially small and completely absent for the subset of articles in journals with impact factors greater than 2.0 and for articles with fewer than 10% decision errors. Nevertheless, it is possible that some aspect of quality could underlie this relation, whether due to the quality of the copy-editing team, better reviewers catching obvious statistical reporting errors or diligence on the part of authors.

More problematic, however, is that some of our analyses indicate that impact factors and citation counts are associated with *poorer* quality. For instance, articles in journals with higher impact factors are associated with *lower* evidentiary value (Question 2) and appear to be less likely to replicate (Question 3)—again noting the considerable uncertainty in the estimates, the magnitude of the *R*_2_, and the BFs. Unlike the presence of statistical reporting errors, these later findings cannot be easily dismissed as typographical errors, as they speak directly to the strength of the statistical evidence and are generally consistent with prior results that show a negative relationship between impact factors and estimates of statistical power in social psychological [34] and cognitive neuroscience [35] journals. Regardless of the causal mechanisms underlying our findings, they converge on the emerging narrative that bibliometric data are weakly, if at all, related to fundamental aspects of research quality (e.g. [4,23–25,34–37,68,69]).

### 6.1. Practical relevance

Promotion and tenure is, fundamentally, a process for engaging in personnel selection, and it is important that it is recognized as such. In personnel psychology, researchers often look for metrics that predict desired outcomes or behaviours without creating adverse impact. Adverse impact occurs when a performance metric inherently favours some groups over others. In this context, a good metric that does not create adverse impact might have a modest correlation with work performance yet not produce differential preferences. Conscientiousness, for example, has a validity coefficient of only about *r* = 0.2 [70], but is widely used because it produces minimal adverse impact [71]. It is questionable that JIFs and citation counts are predictively valid and have minimal potential for adverse impact. As suggested above, citation counts and JIFs likely play an out-sized role in hiring and promotion and for predicting future research productivity (see also [1]). Yet, the research presented above and elsewhere suggests these metrics are over-weighted relative to their utility. There is also plenty of evidence to suggest that they hold the potential to produce adverse impact. Citation counts are known to be lower for women and underrepresented minorities [72–74], and there is some evidence for a negative relationship between impact factor and women authorship [75] and so hiring, tenuring or promoting on their basis may perpetuate structural bias. Second, the present research highlights that the use of these indicators in this context is predicated on the *assumption* that they reflect latent aspects of either research quality or impact. But, inasmuch as the accuracy of statistical reporting (decision errors), evidentiary value (BF) and replication are key components of research quality, our results are inconsistent with this assumption. More problematic, however, is the potential for the *mis*use of these indicators to ultimately select for bad science. Using an evolutionary computational model, Smaldino & McElreath [76] showed that the incentive and reward structure in academia can propagate poor scientific methods. Insofar as impact factors and citation counts are used as the basis for hiring and promotion, our analyses, as well as several other recent findings (e.g. [34,35,69]) are consistent with this model.

The observed inverse relationships between the key indicators of quality on the one hand, and citations and impact factors on the other, have been reported across numerous publications. That is to say, decisions that reward researchers for publishing in high-impact journals and for having high citation counts may actually promote the evolution of poor science [76] as well as select for only certain types of science or scientists [22]. Though our analyses indicate that the consequences might be rather small for any given decision, these selection effects could theoretically accumulate over time.

### 6.2. Limitations and future directions

A rebuttal to our observations that impact factors are either unrelated or negatively related to quality is that our indices of quality (e.g. evidential strength) do not capture other dimensions such as ‘theoretical importance’ or novelty. One can interpret ‘surprisingness’ (see Question 3) as an indicator of novelty, and we are willing to cede that higher-impact journals may well publish more novel research on balance (but see [69] for data suggesting otherwise)—indeed, the ratings of ‘surprisingness’ of studies included in the Open Science Framework replication project were positively, though weakly, associated with impact factors, *b* = 0.151 95% CI = (0.011, 0.293). However, acceptance of this assumption only strengthens our argument. If papers in higher-impact journals are *a priori* less likely to be true (i.e. because they are novel), then it would require more evidence, not less, to establish their validity. Our analyses suggest that the opposite is actually the case. This finding implies the presence of a perverse trade-off in research evaluation: that reviewers and editors for higher-impact journals are more likely to trade strength of evidence for perceived novelty. Regardless of the underlying reasons for this pattern, higher-impact journals are more likely to be read, cited and propagated in the literature, despite the fact that they may be based on less evidence.

We note that each of the datasets we used has certain limitations, although they do not undercut the importance of our results. For example, the program statcheck was used to curate the data for Questions 1 and 2. This program only extracts statistics reported in written text and in APA format (e.g. statistics reported in tables are excluded) and does not differentiate between statistics reported for ‘central’ hypotheses versus more ‘peripheral’ hypotheses, though as we have noted, there is some reason to be cautious in drawing this distinction. A second limitation is the studies included in the replication studies for Question 3 were not a representative sample of many psychology studies. Keeping these limitations in mind, all of our analyses converge on the same point: impact factors and citation counts may be over-weighted relative to their predictive utility.

Several recent working groups have begun drafting recommendations for changes in how the quality of research is evaluated. For instance, Moher *et al.* [32] provide six principles to guide research evaluation. Chief among these principles are the elimination of bibliometric indicators such as the JIF and citation counting, reducing emphasis on the quantity of publications and developing new responsible indicators for assessing scientists that place greater emphasis on good research practices such as transparency and openness. Moher and colleagues’ recommendations also include the need to incentivize intellectual risk taking, the use of open science practices such as data sharing, the sharing of research material and analysis code and the need to reward honest and transparent publication of all research regardless of statistical significance. Some of these recommendations may require fundamental changes in how researchers and administrators view publication.

To conclude, our analysis supports the growing call to reduce the role that bibliometrics play in the evaluation system, and line up with recommendations made in the San Francisco DORA (https://sfdora.org/), the Leiden Manifesto [31] and by numerous scholars [4,32]. More concretely, several researchers have argued that evaluation should focus more on research process and less on outcome, to incentivize behaviours that support open, transparent and reproducible science [32,77,78]. Changing the focus of the evaluation to *how* faculty conduct their work from *what* is produced may shift the incentives to aspects of the research enterprise that is both under the control of the researcher and arguably promotes good scientific practices.

## Data Availability

All of our code and raw data are located on Open Science Framework: https://osf.io/hngwx/.

## Footnotes

1 A further point worth considering is that the distinction between central and peripheral tests may not be so clear. For instance, an exploratory analysis indicating a manipulation check behaved as predicted might initially be regarded as ‘peripheral’, but if the check did not behave as predicted, this test could become more central because it would become an observation the author would need to account for.

2 Although impact factors and citation counts have traditionally been assumed to reflect quality, we anticipated that any observed relationships were likely to be modest at best. The default prior captures this prior belief by weighing small to modest effects more heavily than large effects. Aside from this general justification, we had no other information to guide the setting of the prior.

3 Although this dataset is quite comprehensive, we nonetheless report inferential statistics throughout the paper. This is because even though we have the population of articles for specific years and journals, the statcheck dataset does not reflect the entire population of articles in, for example, cognition, social psychology or clinical psychology. Rather, this dataset is specifically from large publishers like the American Psychological Association, Springer and Taylor & Francis.

4 Analysis of non-decision errors are provided in the electronic supplementary material. Conclusions based on these analyses are generally consistent with those based on the decision errors.

5 One question is whether researchers conceive of citation counts as an index of research quality in the first place rather than as an index of research impact. Replacing ‘quality’ with ‘impact’, raises the question, what is an appropriate index of impact independent of citations? If what we mean by ‘impact’ is societal impact, then it is clear that citation counts are not the right metric prima facie, because citations counts primarily reflect academic, not societal consideration. We suggest that regardless of how else we might define impact, it is bound up with an initial assumption that the research in question is high quality (i.e. replicable, robust and so on).

6 When only considering journals that have impact factors that are two or greater (*N* = 36 499), the relation between JIF and decision errors is diminished, *b* = −0.009 (−0.116, 0.134), BF_10_ = 1/166.67. This suggests that the negative relationship between impact and quality should be interpreted with a considerable amount of caution.

7 To put this in perspective, the Kendall tau rank-order correlation between impact factor and per cent decision errors per paper, an analysis that does not control for the other factors, is inconsequentially small and favours the null (*τ* = −0.002, BF_10_ = 1/142), while the rank order correlation between decision errors and citation counts is also small, the evidence supports a *positive* (Kendall’s *τ* = 0.024, BF_10_ = 9.1 × 10^7^). Adjusting for the necessary grouping and population effects, however, decreases this small bivariate correlation.

8 The log transformation of the BF did not sufficiently correct for the skewness; models other than the beta model or a Gaussian model applied to the rank normal transformation generally failed posterior predictive checks.

9 The inclusion of degrees of freedom also serves as a sanity check, as the BF should scale with sample size, *ceteris paribus.*

10 We also manually looked up each impact factor on the journal website to verify the accuracy of the automated tool. The manually obtained impact factors correlated *r* = 0.99 with impact factors obtained using the Scholar package. We, therefore, report analyses based on the Scholar data. However, we note here that the conclusions based on the manual impact factors are essentially the same.

11 We also conducted analyses using the total number of citations to the senior author of each paper and institutional prestige of the senior author’s institution. The conclusions are unchanged.
